# King Lear in the upper palaeolithic: searching for ethical principles in prehistory

**DOI:** 10.1007/s40592-025-00241-6

**Published:** 2025-04-24

**Authors:** Charles Foster

**Affiliations:** University of Oxford, Oxford, UK

**Keywords:** Atavism, Consciousness, Ethics, Peter hacker, Hunter-gatherer, Identity, Metaphor, Martha Nussbaum, Symbol, Upper palaeolithic

## Abstract

Ethics are concerned with maximising the thriving of individuals and societies. One cannot maximise the thriving of a person unless one has some idea about what sort of creature that person is. Ethics follow ontology. Many answers have been suggested to the question ‘What is a human?’ and the less fundamental question ‘What are the defining attributes of a human’? Many of those answers are theological, and hint that the essence of a human is indefinable; that humans are unknowable, contradictory and mysterious. This article contends that, since behaviourally modern humans have been hunter-gatherers for an overwhelming proportion of their history, we are still foundationally hunter-gatherers, and that accordingly useful insights into our constitution can be gained by examining the quintessential characteristics of Upper Palaeolithic people. Those characteristics are wandering, a relationship with the non-human world, consciousness, story-telling and a consequential ethical sense, a metaphysical instinct, and an operating system based on symbolism and metaphors. Those characteristics (many of which overlap with the conditions of human thriving described by Martha Nussbaum) have ethical corollaries. The article builds on the work of Peter Hacker in contending that it is not only legitimate but necessary to derive ethics from biology and evolutionary history. Humans emerge from this examination, as they emerge from theological speculation and from the work of most creative artists, as unfathomable. The mysteriousness of humans is a reason for according them moral weight. The article suggests that ethicists should take the lead from creative artists, not vice versa.

## Introduction

Ethics are concerned with human thriving (Darr 2017; Foster and Herring 2018; Schnitker et al. 2019).

They are concerned in two different but overlapping ways.

First: Ethics seek to maximise the thriving of individuals.

A clinical example will make the point. In deciding in what individual thriving consists, there is usually – at least in the west – considerable deference to the patient’s own view. If a capacitous patient decides that a surgical procedure is not in her best interests, that is generally regarded as decisive of the question of whether or not the procedure is done. It does not, however, necessarily conclude the question of whether or not it would be in the patient’s objective best interests to have the procedure.

Implicit in respect for patient autonomy is the idea that it is meaningful for an individual to talk about herself as distinct: to see herself as a morally significant entity; a bearer of rights and duties, who is at least in some ways, however socially embedded and defined she perceives herself to be, an *individual.* Put another way, it implies that it is significant and appropriate to use personal pronouns in the way that we usually use them.

Medicine (and particularly psychiatry) explicitly adopts this way of seeing a patient. What is the job of a psychiatrist? It is to the put the patient back in ‘her right mind’. There is, on this view, a proper, normative way of being that patient. Deviation from that way is pathological. This view is at least etymologically coherent: *psychiatrist* means ‘soul doctor’. Psychiatrists, both by name and by their own job description, declare their belief in something akin to a soul. This ‘soul’ is the true substance of the patient: what the patient really is. Psychiatric illness might take from the patient some of the attributes of themselves, but the substance – the ‘soul’ – persists, though compromised, and good medicine can remove the compromise and restore the missing attributes (Foster 2024).

This view of the patient and of the role of medicine rests on the ancient distinction between substance and attributes which goes back to Aristotle and was disinterred in the Middle Ages to defend the doctrine of transubstantiation. How could the Eucharistic bread and wine be truly the body and blood of Jesus, though they tasted like bread and wine? Because, per the doctrine, they were in true substance the body and blood, while having the chemical attributes of bread and wine (Mazza 1999).

I return to this useful and dangerous doctrine throughout this article.

Back to the patient who declines the procedure. It may be objectively in her best interests to have it. If so, a normative assumption about what are the best interests of a person is in play. This assumption will be based on, or at least include, a belief about what a human *as a member of the species Homo sapiens* needs in order to thrive, and such a belief in turn rests on a set of assumptions about what a human *is.*

This brings us to the second sense in which medicine, and ethics more generally, are concerned with human thriving. They are concerned with helping the patient or other subject to thrive objectively – an enterprise which involves making the patient/subject conform to the template of a thriving human. On this view, correct ethics presuppose an accurate ontology.

Suppose a patient consults a surgeon, asking for the surgeon to remove an entirely healthy leg. Should patient autonomy be the only voice to be heard at the discussion about the appropriateness of the operation? Many would think not. Objections to the procedure rest to some degree on the notion that humans *should* have two legs, which in turn (perhaps, or perhaps not, flirting dangerously on the edge of the is-ought gap) rests on the notion that ‘proper’ (and therefore optimally thriving) humans have two legs (Foster and Herring 2017).

In academic discourse, as opposed to in our intuitions, questions of ethics are not commonly founded on discussions of ontology. This article examines the question of what a human is, and moves on to consider what might follow from the answer in terms of objective thriving and thus in terms of ethics.

Petrarch’s *Familiares* records that Petrarch climbed Mount Veritoux and took out a copy of Augustine’s *Confessions.* As it fell open he saw this passage: ‘And men go about to wonder at the heights of the mountains, and the mighty waves of the sea, and the wide sweep of rivers, and the circuit of the ocean, and the revolution of the stars, but themselves they consider not.’ (Bishop 1966, 4.1).

That, today, would be something of an overstatement. Anthropology is a well-recognised discipline. Philosophical anthropology is a well-recognised sub-discipline. Yet anthropologists rarely ask ‘What is a human?’ unless they are physical anthropologists, concerned with human evolution – in which case the answer is framed in terms of the differences between modern humans and non-modern hominins, and thus in terms of the attributes (as opposed to the substance) distinguishing us from our ancestors.

Identification of the quintessential human substance is the core business of philosophical anthropologists. Many, from Anaxagoras to Kant and Hegel, have based their conclusions on a priori assumptions: grand, overarching principles, just as the theologians do.

‘What is man, that thou art mindful of him?’ asks the Psalmist. He purports to supply his own answer. God ‘made [man] a little lower than the heavenly beings, and crowned him with glory and honour.’ (Psalm 8: 4–5: KJV).

It is no answer. It says not what humans are, but where they are in the hierarchy of being. While it is not an ontological answer, it is nonetheless an *ethically* useful answer. Whatever humans are, they are worthy of the respect due to a quasi-divine being.

Similarly for the notion of the *Imago Dei.* To say that humans are made in the image of God says not what they are (for God is quintessentially indefinable), but mandates certain ways of behaving towards humans. Since God is dignified and worthy of respect so, presumably, are humans. Another important corollary is that humans, made in the image of a mysterious God, are themselves mysterious.

A few philosophical anthropologists, however, are anthropologists (or at least phenomenologists) before they are philosophers. That, I suggest, should be a qualification for being taken seriously. For present purposes, Max Scheler can speak for them all. He identified three fundamental essences of humans. We are *Homo sapiens*, smelling of the Athenian Academy, characterized by our ability to reason and to submit to the rule of *logos*. We are *Homo faber*: symbolizing, linguistic, tool-making, environment-manipulating creatures. And we are *Homo religiosus*: trembling creatures, looking fearfully up to the heavens expecting a thunderbolt, or across at a rock infused with agency, and perhaps regretfully back to a golden age when humans were less corrupt.

Scheler himself found it impossible to reconcile these elements. The impossibility led him to conclude that the humans are indefinable, and that any attempt to describe the substance of humans is doomed (Scheler 1972, p. 186). Humans, for him, are not beings but ‘becomings,’: they are amphibious, self-transcending animals, in the no-man’s land between categories.

These elements map fairly well onto the picture of humans I try to paint in this article. Scheler’s conclusion about the essential incalculability of humans accords both with my own conclusion and with the bottom line of the *Imago Dei.*

## An atavistic account of human nature

We have been behaviourally modern *Homo sapiens* (*us)* for around 45,000 years, since the beginning of the Upper Palaeolithic. The boundaries of the Upper Palaeolithic, the Mesolithic and the Neolithic are blurred behaviourally and chronologically. Transitions occurred at different times in different places, (meaning that we must use a broad brush) but, broadly, the hunter-gatherer lifestyle of the Upper Palaeolithic gave way to the more sedentary Neolithic (characterised by dependence on agriculture) from around 10,000 years ago. The agricultural revolution was significantly later in some places. But if we were hunter-gatherers until 10,000 years ago, about 78% of our time as behaviourally modern humans has been spent as hunter-gatherers. Not only was the hunter-gatherer phase the time when our distinctively modern nature first erupted into history, it was the phase in which we have spent overwhelmingly the greater portion of time exploring what it means to be a human.

Palaeolithic diets are popular. They are based on the premise that since our physiology was fashioned by evolution to deal with the exigencies of hunter-gatherer life, we will be healthier and happier if we replicate hunter-gatherer times in our diet (Konner and Eaton 2010). The basic premise is sound: there has been insufficient evolutionary time for major physiological change. The analogy between physiology and human *nature* is sound too (Kuhn et al. 2004). To a first degree of approximation, we are constitutionally hunter-gatherers. We may not look like hunter-gatherers now, but the changes effected since the Neolithic are skin-deep. Modernity is window-dressing. It does not affect what we are, and hence it does not affect how we should act in order to thrive.

I suggest that there are five particularly characteristic aspects of that formative Upper Palaeolithic time. These are: (1) wandering, (2) an intimate relationship with the non-human world, (3) a sense of self (which ignites ethics and mandates story-telling), (4) a sense that the quotidian material world is not all that there is: a metaphysical instinct, and (5) symbolising and the use of metaphor (Foster 2021).

These are not only characteristic, but (I suggest) so enduring and influential that they can be described as constitutional. They are not only attributes, but so foundational that they should be regarded as aspects of substance.

I consider each in turn, briefly and tentatively.

### Wandering

Hunter-gatherers typically need to know and relate to a lot of territory. They need to walk. Stasis is not an option. And at each new place (which is really each new step), they need to be alert – to pay attention; to relate to where they are. Their routine is a constant encounter with novelty, for nothing in the non-human world is ever quite the same as what went before. There are seasonal rotations, but seasons are capricious. Hunter-gatherers are wired up to be themselves in different places, and so to be both self-possessed and to have an intimate, defining relationship with the place in which they are camping at the time.

The physicality of wandering and the strenuousness of hunting and gathering makes unavoidable an awareness of embodiment. It demotes abstraction. The land is more important than mental maps of the land. Iain McGilchrist argues that language itself (regarded by many as one of the defining characteristics of humans) is itself a product of the motion and emotion of the body – a creature of the embodiment that is curated more effectively by wandering than sedentism (McGilchrist 2019). Likewise *reason*, according to Lakoff and Johnson, is ‘not purely literal, but largely metaphorical and imaginative. Reason is not dispassionate, but emotionally engaged… Since reason is shaped by the body, it is not radically free, because the possible human conceptual systems and the possible forms of reason are limited. In addition, once we have learned a conceptual system, it is neurally instantiated in our brains and we are not free to think just anything’ (Lakoff and Johnson 1999; McGilchrist 2021, p. 894). A reasonable human, on this analysis, is not just one who follows an algorithm slavishly (hence so-called artificial intelligence is not really intelligence at all), but one whose engagement with and assessment of the world is embodied, and brings to the algorithms inherent in neural processing the very different, deeper and *wiser* perspective of living as an embodied entity in the world being construed. Embodiment donates to humans most of the element of *sapiens* in our species name. The constraints on our thinking imposed by our embodiment perhaps stop us from going mad – or at least draw the boundaries of what we conventionally think of as sanity.

Implicit in Lakoff’s and Johnson’s view of reasonableness is doubt about the centrality of language to the process of reasoning. We use language to articulate the end result of a chain of metaphorical or imaginative reasoning, but language is unlikely to have started the chain. Embodied reason is surely more likely to have been conceived by media or sensation that operate at a level deeper than language: by music, for instance, or by the surge of hormones caused by ecstatic dance (Mithen 1988, 1990, 1996, 2006; Savage et al. 2021). Dionysus may be more truly reasonable than Apollo. Even now, only a small fraction of our communication with others happens using language: much more uses non-linguistic cues of various kinds (Ruch et al. 2016; Centola 2018). And as to what we as individuals really are? The basic premise of psychoanalysis, nightly confirmed in our dreams, is that most of what we are, and what determines our actions and inclinations, lies well below the level of our consciousness – at a level in which language might stray, but in which language does not rule (Foster 2024). Our ethics and our agency are not determined by the cold examination of syllogisms. This is an embarrassment for professional ethicists. It means, at least, that more serious attention should be given to the role of intuitions in ethical decision-making, and that our early history as a species might indicate how to regard our intuitions (McGee and Foster 2024).

### Relationship with the non-human world

Hunter-gatherers are necessarily superb naturalists, knowing an enormous number of species and an enormous amount about the lives of many species. They, just like us, are wholly dependent on the non-human world for survival but they, unlike us, know it. They are natural ecologists, and typically (and biologically accurately) see the boundaries between themselves and the non-human world porous or non-existent. Upper Palaeolithic shamans transmuted into non-humans: the animal-headed figures on the cave wall of Europe may depict shamans in the process of becoming non-human, or returning (Winkelman 2002; Lewis-Williams 2011). That, though they were not to know it, was a depiction both of the Darwinian truth about human origins, and of the ecological conversation between the human and the non-human worlds. The interconnectedness of humans and non-humans, and the importance of curating that connection, are demonstrated by the growing evidence of a connection between human health and happiness and a relationship with the natural world (Hartig et al. 2014; Franco et al. 2017; Frumkin et al. 2017; Tillmann et al. 2018; White et al. 2019; Lackey et al. 2021).

This orientation towards the non-human world demanded an elaborate liturgical choreography. It made it morally problematic to eat, for eating a fellow or near-fellow required request, oblation and satisfaction (Hill 2011).

It was not until the Neolithic that the human-non-human boundaries lost their porosity. It was in the Neolithic that humans began to see themselves as distinct from the non-human world of which they had previously been – and known themselves to be - an indivisible part. With that new perspective came the notion of hierarchy: humans began to see themselves as better than their subject animals, and (although there were other factors in play - notably those concerned with the practice and economics of grain-growing), this idea perhaps fuelled the development of gender and other hierarchies in human communities too (Scott 2017). Hunter-gatherer communities are typically egalitarian (Cashdan 1980; von Rueden 2020). If women (usually responsible for more gathering than hunting) are providing the lion’s share of the calories, it is harder for men to believe or assert that they are the linch pins of the clan (Deb 2023).

An acknowledgment of relationship – whether with the non-human world or other humans - is necessarily an acknowledgement of dependence. It makes vulnerability an essential element of our ontology.

### Consciousness, ethics and story-telling

Behavioural modernism is the phase when the human sense of self becomes recognisably our own. It is marked by a consciousness of individuality which has come to be regarded (anthropocentrically and probably inaccurately) as consciousness itself. However unsatisfactory this identification of modern human consciousness with consciousness itself, modern human consciousness does have some distinctive footprints (Ingold 1991; Dennett 1998; Donald 2001; Velmans 2015). It is manifested in the explicit or implicit use of personal pronouns. ‘I’ is visible in the archaeological record in depiction of the human form.

‘I’ generates the possibility of the ‘I-Thou’ relationship, which is the root not just of individual and corporate responsibility, and thus of ethics, but of story. The new or newly perceived ‘I’ had to be located in a comprehensible way in relation to the other ‘I’s of the clan, and in relation to the cosmos. The bequest of consciousness - of the ‘I’ – was refined in stories around the campfire. We became creatures who needed to understand themselves and their relationships, and who had an insatiable appetite for *meaning*. Reflection became a defining characteristic, and story became the vehicle of that reflection (Smith et al. 2017). Since stories have a beginning, a middle and an end, a sense of dialectic purpose became infused into human self-understanding.

### A metaphysical instinct

It was a short step from this desire for self-location in a baffling cosmos, and the use of story for that self-location, to an intuition (perhaps initiated or refined by shamanic voyaging to other states of consciousness, thought of as other worlds) that the physical world is not all there is.

There is a striking consensus that (with a minor and bitterly contested caveat about Neanderthal burials) if one sees grave goods, one is dealing with behaviourally modern *Homo sapiens*, and if one does not, one is not. We are quintessentially religious people: *Homo religiosus* before we are *Homo sapiens.*

Paul Pettit convincingly contends that Upper Palaeolithic burials indicate that the dead were seen as possessing *more* agency than the living: that the spirit world was more solid and influential than the world of flint and fur: ‘For most humans [in the Upper Palaeolithic] death is not conceived of as an abrupt end of the individual but a transformation from one state to another, one which usually results in an increase in the power of their agency as they ‘transcend’ the biological world.’ (Pettitt, 2015, 262). Metaphysics trumped physics. Metaphysics were and are more important to human understanding of humans and of the rules of causation than physiology, psychology or thermodynamics.

### Symbolizing and the use of metaphor

The start of the Upper Palaeolithic is marked by an exuberant explosion of symbolism. Things are made to stand for other things. Useful artefacts remain useful artefacts while at the same time becoming visually pleasing (Chase 1994; RielSalvatore and Clark 2001; Bar-Yosef 2002; Jochim 2011).

It is impossible to overstate the importance of metaphor for human operating systems. McGilchrist, building on the work of Lakoff and Johnson, (Lakoff and Johnson 1980, 1999) contends that ‘metaphor is not an embellishment of thought, but its very ground. The form (metaphor) is not added to the thought, but is itself the stuff of thought…’ (McGilchrist 2021, p. 893). He grounds this contention in the argument, mentioned above, that language proceeds from *embodied* human experience. The right cerebral hemisphere – the hemisphere with metaphorical understanding – is the hemisphere that draws its understanding of the cosmos from embodiment rather than abstraction: from the real world rather than a map of the world. Metaphor, then, far from being a self-referential and self-reverential device, enshrining a solipsistic universe, is instead a way of explicating the cosmos based on the real web of relationships linking all things in the world. It is yet another reflection of the interconnectedness of everything; of those basic ecological facts so palpable to hunter-gatherers.

## Some ethical corollaries

While philosophical anthropologists in Scheler’s phenomenological tradition have been ready to draw (usually equivocal) conclusions about human nature from the biological facts, few have drawn any ethical corollaries. This may be a result of fear of the is-ought gap. I address that concern briefly below. But I suspect that it is more likely to be a historical phenomenon. For most of history, morality has been a bequest of God or gods. More recently, when gods were banished from moral discourse, morality has been derived from principles located in a transcendental realm not unlike Olympus – principles such as autonomy, beneficence, non-maleficence and justice. There is rarely any attempt to identify the antecedents or interrogate the authority of these principles (Moncrieff 2022).

Peter Hacker contends that ‘the notion of the good of a being is biologically rooted’ (Hacker 2011). ‘There would be no morality without animality’, he contends. ‘Human nature is the source of many kinds of value’(Hacker 2021). He leans on Von Wright’s taxonomy of species of value to illustrate the point (Von Wright 2004). Hedonic goodness, for Von Wright, is the good of the pleasant; beneficial goodness is what is good for and does good to someone; technical goodness is the goodness of aptitudes; instrumental goodness the goodness associated with implements; and medical goodness the goodness associated with physical wholeness. All of these, argues Hacker, flow, and can be shown to flow, from human nature (Hacker 2021, p. 15), and in particular from characteristics such as our immaturity as infants and our need for nurturing, our sexual desire, our vulnerability to disease, injury and death, our aggression, competitiveness and co-operation, our capacity for imitation and learning, our inclination to pursue goals and follow rules, our fear, curiosity, affection, shame and pride, and, perhaps supremely, our dependency on others for survival and for self-realisation. We are essentially group animals, and our morality determines how we behave in those essential groups. Morality is a quintessential characteristic because it is a biologically essential characteristic. It acts as the social adhesive which keeps the crucial groups together (Moncrieff 2022).

Thus far the argument might sound like an iteration of the familiar reductionist contention that morality is disguised selfishness, endorsed by natural selection because it improves the evolutionary fitness of a gene-bearing individual. But that is not Hacker’s position. Rather, says Hacker, evolutionary imperatives dictate *inclinations*, and the nature of those inclinations mandate moral behaviour to enable the existence and flourishing of social life. Note that this model presupposes a communitarian account of the individual. The individual can only be described in terms of the nexus of relationships in which she exists, and her defining responsibilities described only in terms of that nexus.

Martha Nussbaum has identified ten core human needs, or ‘capabilities’, central to human flourishing (Nussbaum 1997, 2007). The most fundamental is *life–* for all other needs are contingent on it. Then there are bodily health, bodily integrity, use of one’s senses, one’s thought and one’s imagination (including the ability to become attached to something other than oneself), practical reason (including knowing what is good and what is not), affiliation (including the ability to be sympathetic and empathetic), a relationship with non-humans, play, and control over one’s environment (which includes political freedom and access to adequate resources).

This fairly exhaustive list of needs is easily translated into a set of defining human attributes (for we presumably fulfil our needs best, and thrive best, when we act in accordance with our true nature). It is easily translated, too, into an ethical code (for we surely have duties to foster these attributes in ourselves, and a responsibility to strive to ensure that others have and evince them too.

Mention of obligations arising from the way we constitutionally are of course raises the issue of the ‘is-ought gap’ (Lemos 1999). Have I fallen for the naturalistic fallacy? I say no.

Just what Hume’s famous claim about ‘is’ and ‘ought’ entails is bitterly contested. The claim may mean many things, but it means at least that an *ought* can be inferred from an *is* if, and only if, as well as matters of mere fact, one has an evaluative premise (Sayre-McCord 2023).

This is an important and often useful principle. But its utility depends on the context. Where there is an overwhelming consensus about the relevant evaluative principle there is no need to use Hume’s tool. Where there is no consensus, the tool is vital.^1^

In the context of this discussion about human ethics, it seems to me that there is no real dispute about the evaluative principle. One may dispute that every one of Nussbaum’s core needs is indeed a core need, but it cannot and is not seriously disputed that an ethical responsibility arises to help others to possess each capability that is in fact a core need.

I am therefore confident that it is legitimate to derive ethical conclusions by identifying core human attributes. I am reassured, too, by Hacker, that it is not only legitimate but mandatory to derive ethics from human biology. This conclusion will lean towards a natural law analysis of human obligations, but the connection between Hacker and natural law is neither simple nor uncontroversial, and a full exposition of the jurisprudential consequences of Hacker’s view is beyond the scope of this article.

I go further than Nussbaum and Hacker, however. The five characteristics of the first behaviourally modern humans that I have identified are so early in our history and so intimately causally connected with the most fundamental characteristics that we use to describe ourselves that it is appropriate to describe them as different elements of human *substance* rather than mere attributes. They are more akin to the Eucharistic body and blood than to the chemistry of bread and wine.

If they are akin to the substance, one might expect them to lend enhanced authority to their ethical corollaries. But the distinction may not matter. Even if they are ‘mere’ attributes, they are still attributes which are closer to the root of human substance than those in Nussbaum’s or Hacker’s list, and give an older and more fundamental justification for the importance of those attributes.

I need not spell out the corollaries in detail: as noted, many of them reiterate or slightly re-state many of Nussbaum’s characteristics. As for Nussbaum’s characteristics, identification of a fundamental characteristic connotes an ethical obligation to maximise it in oneself and others.

Wandering (perhaps paradoxically at first blush) means that an intimate relationship with place is essential, but that constant change and movement (expressed in curiosity, aspiration, and the possibility of meaningful development) are too.

Embodied reason means both that bodies are important (implying obligations to preserve life and health) and that reason should be a bulwark against caprice and arbitrariness.

A relationship with the non-human world entails a profound ecological concern and an acknowledgment of human dependence on the non-human world (which itself generates humility and an acknowledgment of dependency per se as a fundamental characteristic). Dependency on the non-human world, and the choreography necessary to negotiate that dependency, makes us fundamentally sacramental creatures. The porosity of the membranes separating us from others entails an acknowledgement of relationality as a basic constituent of the world, leading to communitarianism, egalitarianism, a rejection of solipsism, and hence the imperative of justice and the need to ensure a level playing field in the making of decisions.

In consciousness and story-telling are to be found the justification for ethics itself. A proper sense of self implies a sense of the other, which in turn generates personal and corporate responsibility (Bowring 1997; Tam and Tam 1998; Fowler 2004; Smith et al. 2017; Tabensky 2017). The stories we compulsively tell about ourselves and others and our place in the cosmos are all *big*, literally cosmic stories, telling of creatures of great moral weight. Mere moral weight, without more, is a brake on the casual treatment of others, and a mandate for earnest moral deliberation.

Our inalienable metaphysical instinct, similarly, means that we see humans and non-humans as morally significant, and the consequences of our actions and inactions as extending beyond the temporal. It invests matter itself with a significance beyond the utilitarian, so curating a stewardship responsibility towards human and non-human bodies and towards the material world.

The mechanism of our operating system – symbolising and the use of metaphor – makes us incurable recruiters of everything to explain everything else. This works synergistically with our sense that everything is connected, and hence to our communitarianism, our relationality, our dependency and our sense of responsibility for entities other than ourselves. Since, in a symbol and a metaphor, a thing can be both itself and something else, the world is infinitely valent – an insight that breeds wonder, curiosity, respect, and, again, curatorial responsibility. The capacity for metaphor makes us adept tool-makers, and thus boosts our agency and strengthens the ethical strings attached to agency.

## Atavism, mystery and the arts

This is a more mystical account of humans than is usually found in bioethical journals (insofar as *any* account of human nature is found there). It smells more theological than philosophical. Yet that is how we have explicitly seen ourselves for the last 45,000 years and, however materially-reductionist the academic zeitgeist is, how we continue to see ourselves. Some might say that it is simply confused – an abrogation of rigour.

In my defence to that charge I can call formidable witnesses. I have already called Max Scheler. I might call Maximus the Confessor (580–662), who contended that each human was a microcosm of the whole cosmos (Blowers 1997; Louth 2013). It was a vision of the human person with consequences comparable to the *Imago Dei.* To kill a person made in the image of God is tantamount to deicide: to kill a person in whom is the cosmos is an act of cosmicide. Since God is infinitely mysterious, so, as I have already observed, a human made in God’s image is similarly mysterious. Likewise for a human who contains the unfathomable cosmos.

But there are less contentious witnesses. They include, I suggest, almost every creative artist in every medium whom we would see as telling the – or a – truth about the human condition. Each tells of the contradictoriness of human nature; of the impossibility of neat generalisations; of our downright weirdness. Shakespeare is the canonical example. He is regarded as great – by which I mean a truth-teller of rare insight – precisely because none of his characters conform to any template, just as no human conforms to any template (Waugaman 2023). His kings are not consistently kingly; his buffoons are wise; his despots fragile and pathetic. Keats famously wrote to his brothers, on 22 December 1817: ‘….it struck me what quality went to form a Man of Achievement, especially in Literature, and which Shakespeare possessed so enormously—I mean Negative Capability, that is, when a man is capable of being in uncertainties, mysteries, doubts, without any irritable reaching after fact and reason….’ Great descriptions of humans are descriptions of mysteries by writers or other artists who realise that they themselves are mysterious.

A great painter shows the child and the fearful mortal in the face of a jewelled queen; a great poet subverts the very language we use for our conceits.

The humanities, in other words, tell the same mysterious and paradoxical tale about us as those Upper Palaeolithic hunter-gatherers – an observation that lends authority to the ontological and ethical lessons we can learn from those hunter-gatherers.

## Conclusion

In his essay *The Human Being and History*, Scheler wrote: ‘In our ten-thousand year history, we are the first time period in which the human being has become fully and totally ‘problematic’; the first time period in which the human being no longer knows who he or she is, but also *knows that* he or she does not know’ (Scheler 1976, p. 120).

This is surely the root of our ontological uneasiness and our personal and societal neuroses. The ethical consequences are obvious. If we do not know who or what we are, how can we know what it is to thrive, and how we should act to maximise our own thriving and that of others?

Upper Palaeolithic hunter-gatherers, Shakespeare and Leonardo da Vinci can help. They agree with one another about what we are. They can help more than professional bioethicists, because the whole business of rigorous bioethics is directed at classifying the intrinsically unclassifiable. Its premise (of the comprehensibility of humans) is misconceived. Bioethicists will only become truly useful if and when they recognises the mystery and incalculability of humans. I do wonder, though, if, when that happens, we will find that the whole superstructure of bioethics collapses into *King Lear* or the cave paintings of Altamira, and the *Principles of Biomedical Ethics* (Beauchamp and Childress 2001) become a minor footnote to the *Rubaiyat of Omar Khayyam.*

## Data Availability

No datasets were generated or analysed during the current study.

## Footnotes

1 I am grateful to Dave Frame for this way of putting the central issue.
